# Parameters of Metabolic Response to Surgical Trauma Induced via Unilateral Total Mastectomy Associated or Not to Ovariohysterectomy in Dogs

**DOI:** 10.3390/ani13050926

**Published:** 2023-03-03

**Authors:** Sabrina Marin Rodigheri, Felipe Noleto de Paiva, Bruna Fernanda Firmo, Taise Fuchs, Cynthia Bueno Mani, Andrigo Barboza de Nardi

**Affiliations:** 1 School of Health Sciences, Ecoville Campus, Positivo University (UP), Curitiba 81280-330, Brazil; 2 Department of Veterinary Clinic and Surgery, Faculty of Agricultural and Veterinary Sciences, Paulista State University (UNESP), Jaboticabal 14884-900, Brazil; 3 Department of Veterinary Medicine, Federal University of Paraná, Curitiba 80035-050, Brazil; 4 Animal Specialized Animal Medicine Center (CEMA), Batatais 13300-000, Brazil

**Keywords:** oncology, surgical oncology, surgical trauma, mammary tumor, dogs

## Abstract

**Simple Summary:**

This study aimed to assess the intensity of metabolic response to trauma induced via unilateral total mastectomy in female dogs with mammary neoplasia and verify whether concomitant ovariohysterectomy increases the organic response. Surgical trauma reduced serum concentrations of albumin and interleukin-2 but increased blood levels of glucose and interleukin-6 and serum cortisol levels. We conclude that unilateral mastectomy induces significant metabolic alterations, and its joint performance with ovariohysterectomy increases the organic response to trauma. Due to the low number of studies evaluating the inflammatory response induced via oncological surgeries in veterinary medicine, and the high numbers of such procedures, our results can help in decision making regarding the treatment of some surgical cases.

**Abstract:**

Surgical excision of solid tumors is required for local control of neoplasms. However, surgical trauma can stimulate the release of proangiogenic growth factors, suppressing cell-mediated immunity and favoring the development of micrometastases and progression of residual disease. The present study aimed to evaluate the intensity of the metabolic response to trauma induced via unilateral mastectomy in bitches with mammary neoplasia, the consequences of its joint performance with ovariohysterectomy, and their respective effects on the organic response. Two groups of animals were evaluated in seven perioperative moments, namely, unilateral mastectomy (G1) and unilateral mastectomy associated with ovariohysterectomy (G2). Thirty-two female dogs were selected, ten clinically healthy, and twenty-two diagnosed with mammary neoplasia. Surgical trauma reduced serum concentrations of albumin and interleukin-2 but increased blood levels of glucose and interleukin-6 in the postoperative of G1 and G2 patients. Moreover, serum cortisol levels increased after unilateral mastectomy associated with ovariohysterectomy. Our findings allowed us to conclude that unilateral mastectomy induces significant metabolic alterations in female dogs with mammary neoplasms and its joint performance with ovariohysterectomy increases the organic response to trauma.

## 1. Introduction

Mammary neoplasms are commonly diagnosed in female dogs, and surgery is the main therapeutic modality for local control of the disease. Several surgical techniques have been used to treat mammary neoplasms in dogs. The most appropriate method is selected based on size, location, and number of tumors, as well as the occurrence of invasion into adjacent tissues [1,2].

Unilateral mastectomy (UM) enables the removal of macroscopically visible lesions and occult tumors, reducing the risk of new lesions due to the excision of all neoplasia-affected mammary chain tissue [3]. However, removing more tissue than enough to obtain tumor-free surgical margins has proved to be unnecessary, as radical techniques do not promote increased patient survival [4].

Tumor recurrences and metastases are the main failures when treating mammary neoplasms, especially in dogs diagnosed with high-grade or advanced-stage malignant tumors. Thus, adjuvant therapies are required to maximize disease-free time [5]. Sorenmo, Shofer, and Goldschmidt (2000) [6] found that performing ovariohysterectomy (OH) concomitantly with UM increases the survival of female dogs with mammary neoplasia. Kristiansen et al. (2016) [5] showed that OH can be beneficial in dogs diagnosed with grade-2 mammary carcinomas and positive tumor immunostaining for oestrogen receptors.

Furthermore, metabolic response to trauma (MRT) is a set of neuroendocrine and inflammatory changes after injury aimed at re-establishing homeostasis [7]. The main metabolic alterations associated with trauma are an increase in the secretion of pituitary hormones, activation of the sympathetic nervous system, and release of pro-inflammatory cytokines [8]. The intensity of the organic response depends on the magnitude, nature, and duration of the stimuli. Medium and large surgeries can trigger such a large response that it causes a deterioration of the host’s regulatory processes, contributing to postoperative morbidity and mortality [9].

Attenuation of metabolic response to surgical trauma significantly reduces incidence of postoperative complications [10]. Studies in human patients with cancer have shown that adequate analgesia in the perioperative period reduces susceptibility to tumor recurrences and metastases, as pain induces the suppression of cellular immunity [11]. Protocols with inhalational anesthesia combined with analgesia with opioids follow a worse prognosis in cancer patients when compared to protocols using locoregional anesthetic blocks [12]. Studies on humans and laboratory animals have highlighted that opioid analgesics, including morphine and fentanyl, enhance the surgery-induced suppression of cellular immunity. However, studies with tramadol have shown high analgesic potency associated with reduced postoperative immunosuppression [13]. In veterinary medicine, there are no studies on the modulation of metabolic responses to surgical trauma in cancer patients.

When considering cancer patients, surgical trauma-derived alterations may also promote neoplastic development through stimuli of micrometastases [14,15,16]. Additionally, surgical manipulation of tumors can induce the release of tumor cells into the bloodstream, favoring the spread of neoplasms [12,17]. However, studies on neuroendocrine and stress responses induced via oncological surgeries are scarce in veterinary medicine. In this sense, understanding metabolic responses to surgical trauma in cancer patients allows for selecting anesthetic protocols, surgical techniques, and therapeutic measures that reduce perioperative morbidity, risks of local recurrences, and metastases.

Based on the above, our study aimed to evaluate the intensity of metabolic responses associated with surgical trauma induced via unilateral mastectomy in female dogs with mammary neoplasia and verify whether concomitant ovariohysterectomy increases the organic response to trauma.

## 2. Materials and Methods

### 2.1. Animal Selection

Thirty-two female dogs were selected regardless of breed and age, ten clinically healthy, and twenty-two diagnosed with mammary neoplasia. The patients came from a university hospital in the city of Curitiba, Paraná State (Brazil). The study was approved by the Committee on Ethics in Animal Use (CEUA) of the Evangelical College of Paraná (Protocol n° 001333/2013).

Anamnesis, physical examination, laboratory tests (complete blood count; blood glucose concentration; and serum levels of alanine aminotransferase, alkaline phosphatase, urea, and creatinine), and diagnostic imaging tests (abdominal ultrasound and chest X-ray in the right and left ventrodorsal and laterolateral projections) were performed to assess the clinical condition of the patients with mammary cancer and define tumour staging (World Health Organization; Table 1). In patients with multiple tumours, we chose to analyse those with the largest diameter for clinical staging.

Caption: N0 (no metastatic involvement in lymph nodes), N1 (metastatic involvement in lymph nodes), M0 (no distant metastasis), M1 (distant metastasis present), T (tumor size).

Animals that showed no clinical manifestations of systemic diseases, nor changes in physical examination or in laboratory and imaging tests, were considered clinically healthy. Moreover, we excluded females weighing less than 7 kg; pregnant; with other neoplasms; with detectable distant metastases (stage V); carriers of systemic diseases that interfere with blood glucose and serum concentrations of albumin, cortisol, and cytokines; and those which had been medicated with anti-inflammatories or subjected to other surgical procedures within 90 days prior to the study.

Animals were initially split into two groups: a control group with healthy females (n = 10) and a mammary neoplasm group (n = 22). Then, mammary neoplasm patients were randomly distributed into two subgroups as a function of the surgical procedure performed. Eleven females that underwent unilateral mastectomy (UM) were included in group 1, and eleven females that underwent UM associated with ovariohysterectomy (OH) were included in group 2. In females diagnosed with tumours in both mammary chains, the chain with the largest tumours was resected.

### 2.2. Procedure and Parameter Evaluation

Patients in the mammary neoplasm group were evaluated in the immediate preoperative period before preanesthetic medication (M0), at the end of the surgery (M1), three hours after the end of the surgery (M2), six hours after the end of the surgery (M3), twenty-fours hours after the end of the surgery (M4), seventy-two hours after surgery (M5), and ten days after surgery (M6). Patients in the control group were evaluated only at M0 and were not subjected to any surgical procedure. 

At all times of evaluation, we measured heart rate (HR); respiratory rate (RR); systolic blood pressure (SBP); body temperature; and blood levels of glucose, lactate, fibrinogen, albumin, cortisol, IL-2, IL-6, and TNF-α. The clinical parameters were evaluated before the blood collections to minimize the effect of stress on outcomes. SBP was measured using a non-invasive blood pressure device (portable vascular Doppler DV 610V, MedMega, Franca/SP, Brazil).

Before the surgery, animals underwent a twelve-hour food fast and eight-hour water fast. After measuring clinical parameters and blood collections for laboratory evaluations (M0), acepromazine (0.03 mg/kg) and morphine (0.5 mg/kg) were administered via intramuscular as preanesthetic medication. After 20 min, the surgical site was shaved, and the cephalic vein was catheterized for fluid therapy, infusing 10 mL/kg/hour of lactated Ringer’s solution.

Anaesthetic induction was performed with 5 mg/kg propofol intravenously, followed by orotracheal intubation and maintenance with isoflurane diluted in 100% oxygen, using a universal vaporizer. Before the start of the surgery, 5 μg/kg intravenous fentanyl citrate bolus was given, followed by an infusion rate of 0.5 μg/kg/min for intraoperative analgesia. During anesthesia, a multi-parameter monitor, a DX-2010, Dixtal^®^ (Biomédica e Tecnologia, Brasil) was used. After anaesthetic induction, the patients were positioned in dorsal decubitus for subsequent surgical site antisepsis.

In group 2 patients, OH was performed through a median retro-umbilical celiotomy, preceding the UM technique. Then, UM was conducted via elliptical skin incision surrounding the cranial thoracic, caudal thoracic, cranial abdominal, caudal abdominal, and inguinal mammary glands, considering a 2-cm margin of safety to tumours, performing en bloc resection of the mammary chain and inguinal lymph nodes. After the procedure, clinical parameters were measured and blood collected (M1), and then the surgical wound was cleaned and dressed. 

In the immediate postoperative period, all animals were medicated with 3 mg/kg tramadol hydrochloride intravenously, remaining under observation until full recovery from anaesthesia. They were maintained on intravenous fluid therapy with lactated Ringer’s solution and warmed with a blanket and room heater. After measuring clinical parameters and collecting blood samples for M2 and M3, the animals were released, and a follow-up was scheduled for the morning after the procedure (M4).

All dogs were medicated in the postoperative period with 5 mg/kg enrofloxacin orally every twelve hours for ten days, 25 mg/kg dipyrone orally every eight hours for three days, and 3 mg/kg tramadol hydrochloride orally every eight hours for five days. Nonsteroidal anti-inflammatory drugs were not administered in the perioperative period to minimize interference with serum levels of cytokines and acute phase proteins.

The patients returned for surgical dressing removal three days after surgery (M5), and then for suture removal in the presence of proper surgical wound sealing after ten days (M6). At all times of evaluation, the patients were evaluated for the presence of hematoma, oedema, or subcutaneous emphysema around the wound, oedema of the pelvic limbs, and the presence of suture dehiscence.

To minimize the influence of the operative technique and circadian rhythm on the serum levels of cortisol, all surgical procedures were performed by the same surgeon and always in the morning.

Samples of tumour and inguinal lymph nodes were stored for histopathological evaluation, following the classification method reported by Cassali et al. (2013) [18].

### 2.3. Statistical Analysis

For statistical analysis, the data collected were initially subjected to the Anderson–Darling residual normality test and presented as mean ± standard deviations for parametric distribution (Gaussian), or median and minimum and maximum values for non-parametric distribution. The Gaussian distribution parameters at each evaluation time were compared with M0 via an analysis of variance (ANOVA) and post-Dunnett’s test, and then a direct comparison between two groups was performed using the Student’s t test. For the non-Gaussian distribution parameters, comparisons between each evaluation moment and M0 were performed using the Friedman’s test and then Dunn’s test, and the comparison between the two groups was performed using the Mann–Whitney test.

When needed, the data were subjected to logarithmic transformation to fit normal distribution of residuals, establish homogeneity of variances, or reduce asymmetry. Pearson’s test was used to determine correlations for parametric data, and Spearman’s test for non-parametric data. All statistical analyses were performed using the GraphPad Prism19 software, at a 5% significance level.

## 3. Results

### 3.1. Animal Characteristics and Clinical Presentation of Tumors and Postoperative Evolution

Healthy animals (control group) were aged between five and eleven years (6.9 ± 2.28) and weighed between 7.0 and 44 kg (13.18 ± 11.17). Females with mammary neoplasia subjected to UM (Group 1) were between six and fourteen years old (9.4 ± 2.84) and had 8.75 and 35.10 kg (18.92 ± 11.34). Finally, females subjected to UM and OH (Group 2) were between five and fourteen years old (9.4 ± 2.70) and weighed 7.10 and 34.70 kg (17.80 ± 8.31). No significant differences were observed for ages and body weights of patients in the different groups.

As for breed, five female dogs (50%) in the control group were crossbreeds and the remainder comprised the breeds Pug (n = 2; 20%), Scottish Terrier (n = 1; 10%), Cane Corso (n = 1; 10%), and Schnauzer (n = 1; 10%). Ten females (45.5%) with mammary neoplasia were crossbreeds and the remainder were Pit Bull (n = 5; 22.7%), Poodle (n = 2, 9.1%), Cocker Spaniel (n = 2; 9.1%), Dachshund (n = 2; 9.1%), and Rottweiler (n = 1; 4.5%).

At the time of diagnosis, among patients with mammary neoplasia, twenty (95.2%) had more than one tumor, eight (38.1%) had tumors only in the right mammary chain, three (14.3%) only in the left mammary chain, and ten (47.6%) in both mammary chains. Thoracic, abdominal, and inguinal mammary glands were involved in mammary tumors in 11 (52.4%), 19 (90.5%), and 18 (85.7%) female dogs, respectively. Sixteen animals (72.7%) had at least one tumor larger than three centimeters in diameter. Lastly, eight patients (38.1%) showed ulcerated tumors. Of the 22 patients who had mammary tumors, 8/22 (36%) had only malignant tumors; 11/22 (50%) had malignant and benign tumors; and 3/22 (14%) had only benign tumors.

**Figure 1:**
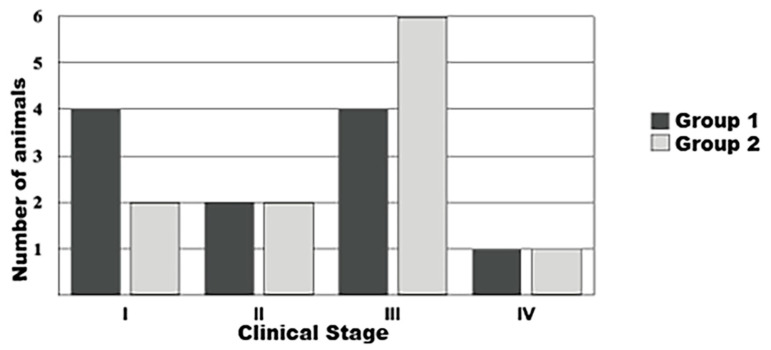
Graphical representation of the clinical staging (stages I, II, III or IV) of mammary neoplasms diagnosed in female dogs subjected to unilateral mastectomy (Group 1, n = 11) or unilateral mastectomy and ovariohysterectomy (Group 2, n = 11). Paulista State Universisty/Jaboticabal (2016).

Regarding mammary tumor clinical staging, six patients (27.3%) presented stage I tumors, four (18.2%) stage II, ten (45.4%) stage III, and two (9.1%) stage IV (Figure 1).

No deaths were recorded in the perioperative period of patients undergoing UM, with or without OH. The main surgical wound-related complications were: hematoma (n = 11; 50%), pelvic limb oedema (n = 5; 22.7%), seroma (n = 4; 18.2%), subcutaneous emphysema (n= 4; 18.2%), and partial suture dehiscence (n = 2, 9.0%). In all animals, skin sutures could be removed on the tenth postoperative day due to the adequate sealing of surgical wound edges.

### 3.2. Histopathological Features of Tumors

Histopathological evaluation of mammary neoplasms revealed malignancy in 86% of patients. Malignant tumors were classified as: carcinoma in situ (n = 11; 31.4%), carcinoma in mixed tumors (n = 11; 31.4%), squamous cell carcinoma (n = 4; 13.6%), papillary carcinoma (n = 3; 8.6%), tubular carcinoma (n = 2; 5.7%), anaplastic carcinoma (n = 2; 5.7%), solid carcinoma (n = 1; 2, 9%), and mammary osteosarcoma (n = 1; 2.9%). As for tumor malignancy grade, 70.6% of carcinomas were classified as grade one, 23.5% as grade two, and 5.9% as grade three. Two animals had metastases in inguinal lymph nodes at the time of surgery, which were diagnosed as grade three anaplastic carcinoma and grade two tubular carcinoma, respectively. Benign or non-cancerous neoplasms were classified as: benign mixed tumor (n = 10; 42.1%), atypical ductal hyperplasia (n = 7; 31.6%), adenoma (n = 5; 21.1%), and ductal papilloma (n = 1; 5.2%).

### 3.3. Clinical and Laboratory Parameters

Table 2 and Table 3 respectively detail the clinical and laboratory outcomes in healthy and diseased female dogs at M0. Higher blood levels of glucose, lactate, fibrinogen, and IL-6 were found in patients with mammary tumours (*p* < 0.05).

**Figure 2:**
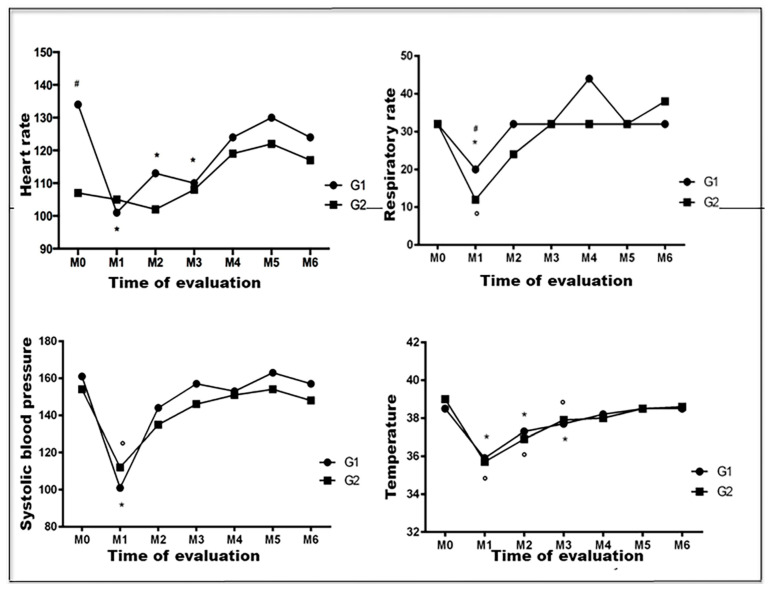
Graphic representation of means (heart rate and systolic blood pressure) and medians (respiratory rate and temperature) of clinical parameters of female dogs with mammary neoplasia subjected to unilateral mastectomy (group 1; n = 11) or unilateral mastectomy and ovariohysterectomy (group 2; n = 11), evaluated at different times of the perioperative period. G1: group 1. G2: group 2. *: significantly different in relation to M0 (group 1). °: significantly different in relation to M0 (group 2). ^#^: significant difference between groups 1 and 2 (*p* = 0.001). Paulista State Universisty/Jaboticabal (2016), Brazil.

Figure 2 graphically represents the results of clinical parameters evaluated at different times in the perioperative period of female dogs with mammary neoplasia subjected to UM, associated or not with OH.

In group 1 patients, HR was significantly lower at M1, M2, and M3 than at M0 (*p* = 0.0121). However, the values remained within the physiological limits for canine species.

In group 1 and 2 patients, RR and SBP were significantly low at M1 (*p* < 0.05). Four animals (36.4%) in group 1 and nine (81.4%) in group 2 had bradypnea at the end of the surgery. Group 2 patients showed a higher reduction in RR compared to group 1 patients at M1 (*p* = 0.001). As for SBP, none of the patients assessed at M0 had hypotension.

Animals from both groups showed hypothermia at M1, M2, and M3, which was more pronounced at the end of the surgery. The association of OH with UM had no influence on the intensity of body temperature reduction.

**Figure 3:**
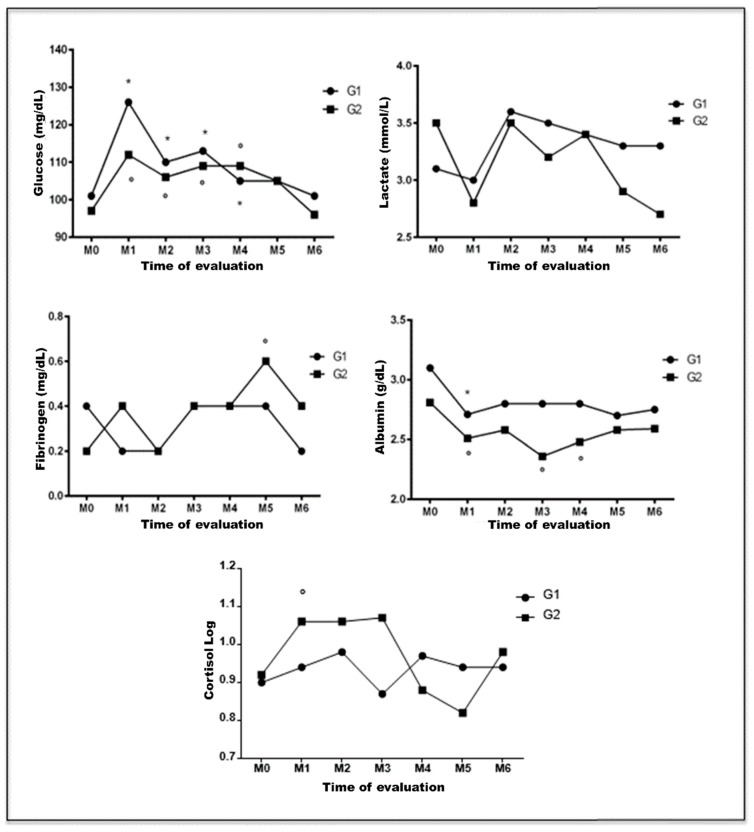
Graphical representation of mean (lactate and fibrinogen) and median (glucose, albumin, and cortisol) values of laboratory parameters of canine females with mammary neoplasia subjected to unilateral mastectomy (group 1; n = 11) or unilateral mastectomy and ovariohysterectomy (group 2); n = 11) at different times in the perioperative period. G1: group 1. G2: group 2. *: significantly different in relation to M0 (group 1). °: significantly different in relation to M0 (group 2). Paulista State Universisty/Jaboticabal (2016), Brazil.

Figure 3 graphically represents the blood levels of glucose, lactate, fibrinogen, albumin, and cortisol measured at different times during the perioperative period.

Both group 1 and 2 patients had glycaemia increases significantly higher at M1, M2, M3, and M4 when compared to that at M0 (*p* < 0.05). Seven animals (63.6%) of group 1 and six (54.5%) of group 2 had hyperglycaemia after surgery. At M6, glycaemic levels returned to values similar to those recorded preoperatively.

No changes were observed in blood lactate levels at all evaluation times for both groups (*p* > 0.05). However, a significant increase in plasma fibrinogen was recorded at M5 for female dogs under UM associated with OH (*p* = 0.0238), with hyperfibrinogenaemia being evidenced in six animals (54.5%).

Serum albumin concentrations were significantly lower at M1, M3, and M4 compared to that at M0 in group 2 patients (*p* = 0.0100). At M2, four animals (36.4%) had serum levels below 2 g/dL. Group 1 patients showed reduced concentrations of albumin only at M1 when compared to M0 (*p* = 0.0300). Despite the lack of significant difference between groups 1 and 2, hypoalbuminemia was evidenced at all times during the postoperative period for animals subjected to UM associated with OH.

Although an increase in serum cortisol concentrations was observed at M1, M2, and M3 in group 2 patients, a significant difference was found only at M1 compared to M0 (*p* = 0.0492).

**Figure 4:**
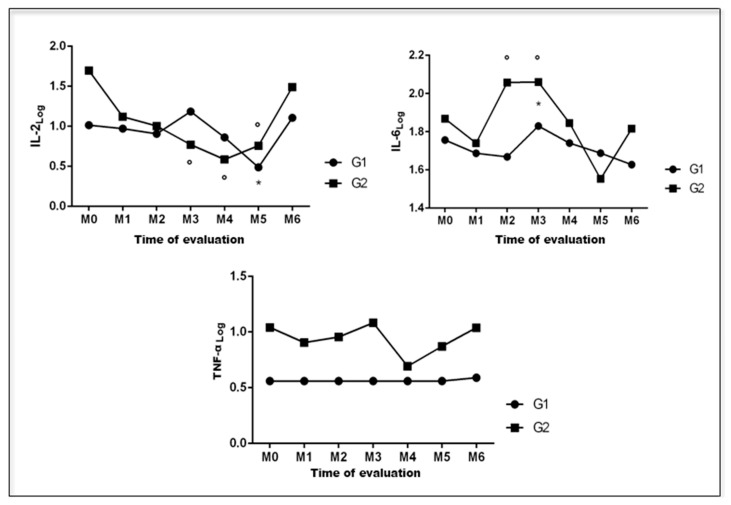
Graphical representation of median values of serum concentrations of IL-2, IL-6, and TNF-α in canine females with mammary neoplasia subjected to unilateral mastectomy (group 1; n = 11) or unilateral mastectomy and ovariohysterectomy (group 2; n =11) at different times in the perioperative period. G1: group 1. G2: group 2. *: significantly different in relation to M0 (group 1). °: significantly different in relation to M0 (group 2). Paulista State Universisty/Jaboticabal (2016), Brazil.

Figure 4 graphically represents the serum levels of IL-2, IL-6, and TNF-α of female dogs with mammary neoplasms at different times during the perioperative period.

Serum concentrations of IL-2 significantly decreased at M5 for group 1 patients (*p* = 0.0465), and at M3, M4, and M5 for group 2 (*p* = 0.0187). Serum concentrations of IL-6 increased significantly at M3 for group 1 patients (*p* = 0.0035), and at M2 and M3 for group 2 patients (*p* = 0.0035). At M4, they returned to preoperative levels in both groups. As for TNF-α blood levels, no significant changes were observed at all evaluation times for groups 1 (*p* = 0.1105) and 2 (*p* = 0.4555). Furthermore, no correlations were observed between the evaluated parameters and clinical stage of tumors, or even the duration of surgeries in patients with mammary neoplasia.

**Figure 5:**
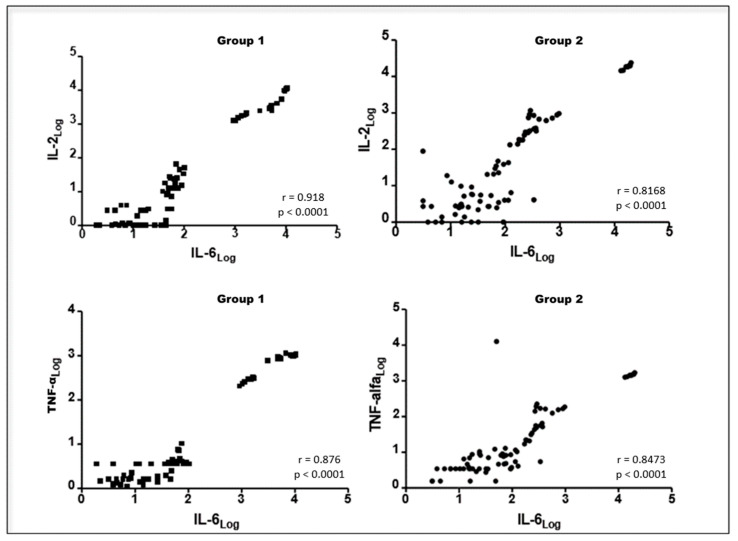
Graphical representation of the Spearman correlation between serum concentrations of IL-6 and IL-2, IL-6, and TNF-α in canine females with mammary neoplasia subjected to unilateral mastectomy (group 1; n = 11) or unilateral mastectomy and ovaryhysterectomy (group 2; n = 11) at different times in the perioperative period. Paulista State Universisty/Jaboticabal (2016), Brazil.

**Figure 6:**
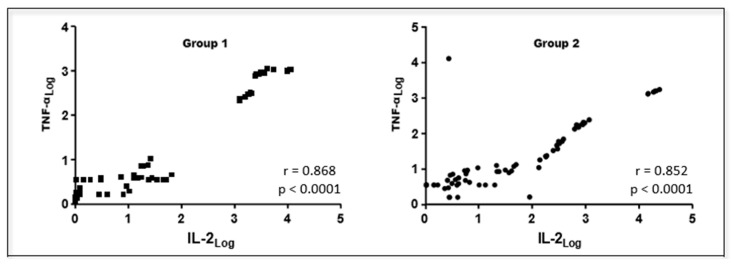
Graphical representation of the Spearman correlation between serum IL-2 and TNF-α concentrations in canine females with mammary neoplasms subjected to unilateral mastectomy (group 1; n = 11) or unilateral mastectomy and ovariohysterectomy (group 2; n = 11) at different times in the perioperative period. Paulista State Universisty/Jaboticabal (2016), Brazil.

We found a positive correlation between blood levels of fibrinogen and serum concentrations of IL-6 (r = 0.256; *p* < 0.05), but a negative correlation between serum concentrations of albumin and IL-6 (r = −0.298; *p* < 0.01) in patients undergoing UM and OH jointly. Positive correlations were also observed among serum concentrations of IL-6, IL-2, and TNF-α in group 1 and group 2 patients (Figure 5 and Figure 6, respectively).

## 4. Discussion

Surgical resection is the therapy of choice for mammary tumors, as it provides the greatest chance of cure [1,2]. However, studies have suggested that surgical trauma can facilitate the progression of minimal residual disease and development of metastases, mainly due to the increased production of angiogenic growth factors and suppression of cellular immunity [11,12,17].

Several studies in human medicine have investigated the occurrence and intensity of neuroendocrine and immunological changes in the perioperative period of patients undergoing oncological surgeries [16,19,20,21,22]. However, in veterinary medicine, most studies have been limited to cortisol changes in dogs subjected to ovariohysterectomy, laparoscopic, or orthopaedic surgeries [23,24,25,26]. In this sense, our study enables for understanding the metabolic response to trauma induced via unilateral mastectomy in female dogs with mammary neoplasia, evaluating neuroendocrine and inflammatory parameters.

Higher blood glucose and lactate concentrations were found in females with mammary neoplasia than in healthy females. Increased blood glucose often results from hepatic neoglucogenesis due to high glucose uptake by tumor cells, producing lactate, even under adequate tissue perfusion. Recent studies have hypothesized that lactate may act as a signaling molecule for tumor angiogenesis, thus facilitating the progression of neoplasms [27].

Inflammation has been correlated with tumor invasion and poor prognosis in women with mammary neoplasia. Besides acting in the immune and inflammatory responses, cytokines play major roles in tumor initiation, growth, and metastasis [28]. In our study, female dogs with mammary cancer had higher serum levels of IL-6 than healthy ones. In this context, Ravishankaran and Karunanithi (2011) [29] observed a correlation between IL-6 concentrations and tumor staging in women with breast carcinoma, with higher levels in patients with detectable metastases. Conversely, we found no correlation between serum IL-6 concentrations and tumor staging.

Plasma fibrinogen concentrations were also higher in females with mammary neoplasia. It may be because IL-6 stimulates protein production in the acute phase [8,30]. We observed hyperfibrinogenaemia in 22.7% of patients with mammary cancer in the preoperative period. Studies on men and women diagnosed with solid tumors have revealed that preoperative hyperfibrinogenaemia is associated with tumor progression, thus having a negative correlation with survival time [31,32]. Andreasen et al. (2012) [33] found that animals with detectable metastases had significantly higher plasma concentrations of fibrinogen. This could not be verified in our study due to the small number of patients with metastases in lymph nodes and the exclusion of females with distant metastases.

The intensity of metabolic responses to surgical trauma can be influenced by different factors, including the extent of the surgery, surgical technique used, surgery length, and anesthetic protocol [7,10,20,34,35,36]. 

UM is considered a highly invasive surgery due to incision and tissue resection extents, thus inducing moderate to severe pain [37]. According to Krikri et al. (2013) [38], the intensity of abdominal surgery-related trauma is determined by the size of incisions in the skin, subcutaneous tissue, aponeuroses, and parietal peritoneum, in addition to the handling of viscera in the abdominal cavity. In OH, ovarian pedicle traction stands for the moment of greatest nociceptive stimulus [23]. Due to the greater tissue trauma and nociceptive stimulus induced by performing UM and OH concomitantly, our goal was to compare MRT parameters of female dogs undergoing UM and those under UM associated with OH. The mean time of UM associated with OH was significantly longer than that of UM alone; however, no correlation was observed between surgical time and MRT.

MRT begins preoperatively when the sympathetic nervous system (SNS) is stimulated by fear and anxiety associated with fasting, hospital stay, and handling for preanesthetic medication. Moreover, anesthetic induction and orotracheal intubation also stimulate release of catecholamines [7,39]. In the hypodynamic phase, which begins immediately after tissue trauma, hypotension, hypothermia, and tachycardia may occur as a result of the reduced circulating blood volume [40,41]. We noted reductions in blood pressure, bradypnea, and hypothermia at the end of both surgical procedures, but such changes might be related to the anaesthetic protocol.

Acepromazine, which is used in preanesthetic medication (PAM), produces relevant effects on the cardiovascular system due to myocardial depression and the blockade of peripheral α-1 adrenergic receptors [42]. Volatile anesthetics cause a dose-dependent reduction in blood pressure, which is related to a drop in systolic volume or peripheral vascular resistance [43]. In dogs anesthetized with isoflurane, acepromazine tends to reduce mean arterial pressure by 24%, lasting between two and three hours. It, thus, justifies the drop in systolic blood pressure at the end of the surgery in both groups evaluated. Heart rate is not significantly altered after PAM with acepromazine and inhalational anesthesia with isoflurane [43], as we observed in group 2 patients.

Propofol is a sedative and hypnotic used for anaesthetic induction, with a rapid onset of action that lasts only 20 min. It may transiently reduce blood pressure and myocardial contractility, in addition to inducing apnoea [43]. Therefore, the effects observed at M1 for both groups may not be a consequence of its use, since its action is shorter than surgery lengths.

Significant reduction in RR at the end of the surgery in both groups may be due to administration of morphine as a preanesthetic medication, continuous infusion fentanyl in the transoperative period, and isoflurane for anesthesia maintenance. Morphine is an opioid analgesic with a high affinity for µ receptors, which has as main effects: sedation, analgesia, and respiratory depression. Fentanyl is a µ-agonist opioid analgesic with high potency, but it can cause bradycardia and respiratory depression [43,44]. Inhalation anesthesia with isoflurane causes respiratory depression associated with central (medullary depression) and peripheral (intercostal muscle dysfunction) mechanisms, in addition to a depression of the physiological ventilatory response to hypoventilation [45]. We noticed that RR decrease was more severe in females under UM and OH in association, which might have been due to the greater respiratory depression induced by a longer exposure to isoflurane [46].

Body temperature reduction in both groups after surgery may be associated with loss of heat via convection due to wide trichotomy, evaporation via humidifying solutions on the body surface during antisepsis, exposure of tissues and body cavities in the intraoperative period, and hypometabolism associated with the hypodynamic phase of trauma. Moreover, hypothermia may have resulted from an impaired thermoregulation due to the use of acepromazine in preanesthetic medication and trans- and post-operative opioid analgesia, in addition to a low heat production associated with the depression of metabolism via inhalational anesthesia [41,47].

Mild-to-moderate hypothermia causes changes in hemodynamics, ventilation, and oxygenation. However, severe cases can lead to metabolic acidosis, arrhythmias, electrolyte disturbances, blood hyperviscosity, hyperglycaemia, and resistance to insulin action [47]. Despite the use of a thermal blanket during surgery, seven patients (31.8%) had body temperatures below 35.5 °C at the end of UM, associated or not with OH, requiring additional artificial heating. In 63.6% of patients, it returned to a physiological standard six hours after surgeries, normalizing in all patients twenty-four hours after surgeries. Therefore, maintaining normothermia is essential to reducing the extent of MRT [48].

In the hyperdynamic phase of trauma, there is an intense mobilization of energy and protein substrates with tachycardia, tachypnoea, and fever secondary to activation of the SNS, as well as an increased secretion of pituitary hormones and production of proinflammatory cytokines [40,41]. In our study, plasma lactate concentrations did not increase at any evaluation times, possibly due to adequate tissue perfusion during the trans- and post-operative periods [41]; however, they remained above the reference range for dogs at M2, M3, M4, M5, and M6 in group 1 patients, and at M2, M3, and M4 in group 2 patients. Such an outcome may be a result of the greater energy demand in the hyperdynamic phase of MRT, resulting in anaerobic glycolysis [41].

Floriano et al. (2010) [49] reported that lactate production may decrease in the intraoperative and immediate postoperative periods due to a reduction in the peripheral vascular resistance induced via isoflurane, with consequent oxygen delivery. Although some studies have reported that high lactate levels in surgical patients indicate higher postoperative morbidity and mortality [50], others have shown that this substrate is a crucial source of energy, in addition to helping in the tissue repair process by stimulating angiogenesis and collagen deposition [51].

Increased serum concentrations of catabolic hormones after trauma, including cortisol, glucagon, and catecholamines, induce hepatic gluconeogenesis and resistance to the peripheral action of insulin, with consequent hyperglycemia [7], of which the intensity reflects the severity of injuries [48]. In our study, glycemia increased at M1, M2, M3, and M4 in patients undergoing UM, with or without OH; overall, at the end of the surgery, hyperglycemia was recorded in 63.6% of group 1 patients and 54.5% of group 2 patients. A proper glycemic control in the perioperative period is known to reduce mortality rates and postoperative infection [45].

Serum cortisol levels have been widely used to assess pain or postoperative stress in humans and animals [24,25]. Our study showed increased serum cortisol levels after UM associated with OH, but not in patients who underwent UM exclusively. Fox et al. (1994) [23] reported that increased cortisol in the perioperative period is related to the severity of surgical trauma and is more pronounced in abdominal and thoracic surgeries than in surgical procedures on the body surface. Moreover, a neuroendocrine response to surgical trauma is mediated by nervous impulses from the traumatized area, which can be minimized via locoregional anesthesia or total intravenous anesthesia with opioid analgesia [7,10,52].

Inhalational anesthesia does not block the neuroendocrine response to surgical trauma [8]. However, in our study, the administration of morphine in preanesthetic medication, associated with continuous infusion of fentanyl during surgery, may have reduced it in the early postoperative period [7]. Moreover, serum cortisol decreases associated with an absence of tachycardia and tachypnoea in the following evaluations suggest an adequate nociceptive control, although patients’ behavior was not assessed. In this regard, Teixeira et al. (2013) [37] pointed out that tramadol administration at 3 mg/kg every eight hours in the postoperative of female dogs under UM, as performed in our study, can control pain properly.

Zografos et al. (2009) [53] found increased blood glucose and serum concentrations of cortisol and growth hormone in women undergoing excisional biopsy of a mammary tumor under local anaesthesia, both during surgery and at the end of the surgical procedure. In another study with women undergoing mastectomy, serum glucose and cortisol levels four hours after the end of the surgery were higher in patients receiving opioid analgesia than in those subjected to paravertebral anaesthetic block [54]. Still, Horta et al. (2015) [55] did not observe neuroendocrine changes before anaesthetic induction or after orotracheal intubation in dogs undergoing regional mastectomy and UM; however, two hours after the end of the surgeries, there were significant increases in blood glucose and serum cortisol concentrations in patients undergoing UM [55].

Alterations in serum levels of cytokines and acute-phase proteins reflect the presence and intensity of an inflammatory process after trauma, helping the prognosis of patients undergoing different surgical interventions [8,56,57]. Under physiological conditions, concentrations of cytokines are extremely low and may even be undetectable. Changes in plasma cytokine levels may occur within two to four hours after surgery, while in the production of acute-phase proteins these changes occur after twelve to twenty-four hours and may persist for several days, depending on the extent of the trauma [30,58].

Therefore, we observed an increase in the plasma concentration of fibrinogen three days after surgical trauma in females of group 2, possibly due to a greater inflammatory response associated with trauma induced via OH in association with UM. Fibrinogen is not routinely evaluated as an acute-phase protein in dogs and cats due to its late and low-intensity response, as observed in our study [59]. However, in cancer patients, hyperfibrinogenemia may accompany blood hypercoagulability and progression of the neoplastic disease, shortening survival time for patients [31,32,33].

We found a reduction in serum albumin levels after surgery in both groups; however, patients under UM associated with OH had hypoalbuminemia throughout the postoperative period. Reductions in circulating levels of albumin in surgical patients may be due to low hepatic production induced by the acute-phase reaction, blood dilution secondary to intraoperative intravenous fluid therapy, and loss of proteins to the interstitial space [60]. Hypoalbuminemia has been considered a risk factor in surgical patients, following a poor prognosis; the main postoperative complications associated with it include oedema, delayed surgical wound healing, and infection [61]. In our study, plasma fibrinogen concentrations and serum IL-6 levels displayed a positive correlation, while serum albumin levels and serum IL-6 levels exhibited a negative correlation; therefore, an acute-phase reaction occurred in the postoperative period of UM, with or without OH.

IL-6 is the main mediator of an inflammatory response secondary to surgical trauma, and its serum concentrations are related to the extent of the tissue injured and postoperative complications [8,57]. For these reasons, several studies have been aimed at evaluating the serum levels of IL-6 and acute-phase proteins in human patients undergoing oncological surgeries [20,21,22]. Here, we evidenced an increase in serum IL-6 levels at M2 and M3 in group 2 patients and at M2 in group 1 patients, with no difference between groups. Okamura et al. (2015) [22] described a positive correlation between serum IL-6 levels and systemic inflammatory response syndrome (SIRS) duration in the postoperative period of patients with esophageal tumors undergoing esophagectomy.

In turn, IL-2 stimulates the production of T lymphocytes and immunoglobulins, acting on antitumour immunity [62]. Therefore, the reduction in IL-2 circulating levels observed at M5 in patients under UM, and at M4 and M5 for dogs undergoing UM and OH, can lead to the suppression of cellular immunity, facilitating tumour recurrences and metastases [28]. The main cause of reduced serum IL-2 levels after trauma is an increase in prostaglandin E2 concentrations [58]. In our study, serum levels of TNF-α did not change at the different times of the postoperative period in both evaluated groups, possibly due to its half-life being less than 20 min [62].

Horta et al. (2015) [55] showed that postoperative complications are common in female dogs undergoing mastectomy and more often after radical surgical techniques. We found hematomas in 50% of animals, with pelvic limb oedema, seroma, subcutaneous emphysema, and partial suture dehiscence at lower incidences. Hematomas result from transoperative injuries to the complex vascular system of the mammary glands, while pelvic limb oedema may be related to lymphatic drainage damage after surgical removal of the inguinal lymph nodes. In turn, subcutaneous emphysema and seroma formation stem from the dead space formed after resection of the mammary chain. Finally, suture dehiscence occurs in the presence of seroma, infection, and ischemic necrosis at the edges of the surgical wound [63].

We observed a low incidence of seroma and subcutaneous emphysema, which can be attributed to the reduction in the dead space achieved through the use of movable sutures during the transoperative period and application of a compressive bandage in the first three postoperative days. Vitug and Newman (2007) [64] indicated that the main postoperative complications associated with mastectomy in women include: seroma formation, hematomas, surgical wound infection, chronic incisional pain, and venous thromboembolism, with surgeries considered of low morbidity, as observed in our study.

Different studies have compared the benefits of radical surgical procedures for the removal of mammary neoplasms with the conservative excision of tumours [3,4]. Researchers have concluded that radical surgical techniques do not promote longer survival times than conservative techniques since tumour excision promotes surgical margins free of neoplastic cells [4].

Evaluating 99 female dogs with isolated mammary tumors, Stratmann et al. (2008) [3] showed that 58% of animals undergoing regional mastectomy developed a new tumor in the remaining ipsilateral mammary gland, requiring a new surgical intervention. These authors emphasized that UM enables removing macroscopically visible lesions and occult tumors, reducing the risk of new lesions due to the resection of all mammary tissue in the chain affected by the neoplasm. However, Horta et al. (2015) [55] reported that, although unilateral mastectomy can prevent the development of new mammary tumors, it should not be performed prophylactically, as it is an invasive surgical technique that results in surgical stress and postoperative complications.

Although some studies have revealed that performing OH concomitantly with mammary tumor resection can bring benefits to some females with mammary neoplasms [5,6], most traumatic surgical interventions may lead to aggravated organic responses in the postoperative period. We found that unilateral mastectomy induces significant metabolic changes in bitches with mammary neoplasia, and these changes are intensified with concomitant ovariohysterectomy. Thus, additional studies on the modulation of the MRT in female dogs with mammary neoplasia are needed, aiming to minimize the incidence of postoperative complications, such as tumor recurrences and metastases associated with postoperative immunosuppression.

## 5. Conclusions

An analysis of the outcomes allows us to conclude that female dogs with mammary neoplasia have metabolic changes in the preoperative period possible due to tumor development and progression, including increased blood concentrations of lactate, glucose, fibrinogen, and interleukin-6.

We may also conclude that, even with general inhalational anesthesia associated with perioperative opioid-based analgesia, unilateral total mastectomy induces significant metabolic alterations in female dogs with mammary neoplasia, which may be enhanced when in concomitance with ovariohysterectomy. Thus, this fact must be included among the numerous criteria considered in decision making for surgical intervention.

## Figures and Tables

**Table 1:** Clinical stage of mammary neoplasms in dogs.

Stage	Tumor Size	Lymph Nodes	Distant Metastases
I	T1: <3 cm	N0	M0
II	T2: 3–5 cm	N0	M0
III	T3: >5 cm	N0	M0
IV	Any T	N1	M0
V	Any T	N0 or N1	M1

**Table 2:** Clinical parameters [mean ± standard deviation or median (minimum–maximum)] of healthy females (control group) and females with mammary neoplasia evaluated at M0. Paulista State Universisty/Jaboticabal (2016), Brazil.

Parameter	Control Group (n = 10)	Mammary Neoplasm Group (n = 22)	*p*-Value	Normal Values (Adult Dog)
HR (bpm)	132 ± 27.51	121 ± 23.15	*p* = 0.2301	60–120
RR (mpm)	43 (24–80)	32 (12–90)	*p* = 0.1652	18–36
SBP (mmHg)	148 ± 32.59	158 ± 26.80	*p* = 0.3917	80–120
T (°C)	148 ± 32.59	38.7 (38.1–39.7)	*p* = 0.4258	37.5–39.2

HR: heart rate; RR: respiratory rate; SBP: systolic blood pressure; T: body temperature.

**Table 3:** Laboratory parameters [mean ± standard deviation or median (minimum–maximum)] of healthy females (control group) and females with mammary neoplasia evaluated at M0. Paulista State Universisty/Jaboticabal (2016), Brazil.

Parameter	Control Group (n = 10)	Mammary Neoplasm Group (n = 22)	*p*-Value	Normal Values (Adult Dog)
Glucose (mg/dL)	85 (76–98)	98 (73–204) ^b^	*p* = 0.0483	65–118
Lactate (mmol/L)	2.18 ± 0.56	3.3 ± 0.94 ^a^	*p* = 0.0017	1.20–3.10
Albumin (g/dL)	2.95 (2.50–3.60)	2.93 (1.51–3.66)	*p* = 0.7565	2.6–3.3
Fibrinogen (mg/dL)	0.2 (0.2–0.2)	0.3 (0.2–1.0) ^b^	*p* = 0.0109	2.0–4.0
Cortisol (ng/mL)	4.00 (1.08–79.11)	8.08 (2.97–57.13)	-	X
Cortisol_Log_	0.59 (0.03–1.90)	0.90 (0.47–1.75)	*p* = 0.4385	X
IL-2 (pg/mL)	1.15 (1.02–759)	22.64 (0.40–24.134)	-	X
IL-2_Log_	0.06 (0.01–2.88)	1.35 (0.01–4.38)	*p* = 0.0996	X
IL-6 (pg/mL)	4.06 (1.18–469)	63.25 (3.13–20.182)	-	X
IL-6_Log_	0.60 (0.07–2.67)	1.80 (0.58–4.30) ^b^	*p* = 0.0416	X
TNF-α (pg/mL)	2.43 (1.06–216)	4.85 (1.19–1.900)	-	X
TNF-α_Log_	0.38 (0.02–2.33)	0.68 (0.07–3.23)	*p* = 0.1725	X

^a^ significant difference (*p* < 0.05) in relation to the control group using Student’s *t* test; ^b^ significant difference (*p* < 0.05) in relation to the control group using the Mann–Whitney test.

## Data Availability

Not applicable.
